# Serotonin-endocannabinoid crosstalk selectively regulates inhibitory GABAergic inputs in the medial prefrontal cortex

**DOI:** 10.1038/s41386-026-02364-8

**Published:** 2026-02-11

**Authors:** Rodrigo C. Meza, Koyam Morales-Weil, Carlos Ancatén-González, Angélica P. Escobar, Alejandro Alcaino, Nicole Sanguinetti, Eric Delpire, Pablo R. Moya, Chiayu Q. Chiu, Marco Fuenzalida, Andrés E. Chávez

**Affiliations:** 1 Instituto de Neurociencias, Universidad de Valparaíso, Valparaíso, Chile; 2 Instituto de Fisiología, Facultad de Ciencias, Universidad de Valparaíso, Valparaíso, Chile; 3 Centro Interdisciplinario de Neurociencia de Valparaíso (CINV), Universidad de Valparaíso, Valparaíso, Chile; 4 Centro de Neurobiología y Fisiopatología Integrativa (CENFI), Facultad de Ciencias, Universidad de Valparaíso, Valparaíso, Chile; 5 Department of Anesthesiology, Vanderbilt University Medical Center, Nashville, TN USA; 6 Present Address: Facultad de Ciencias de la Vida, Universidad Viña del Mar, Viña del Mar, Chile; 7 Present Address: Department of Neuroscience, Yale University School of Medicine, New Haven, CT USA

**Keywords:** Inhibition, Neurophysiology

## Abstract

Serotonin (5-HT) plays an important role in shaping brain network dynamics by regulating excitatory synaptic function and neuronal excitability. However, much less is known about how 5-HT tunes synaptic inhibition. Here, we demonstrate that transient 5-HT signaling persistently suppresses GABAergic synapses onto layer 2/3 pyramidal neurons in the medial prefrontal cortex (mPFC). Moreover, we found that 5-HT1A and 5-HT2A receptors differentially contribute to 5-HT regulation of synaptic inhibition, possibly by acting at distinct GABAergic cell subpopulations. Importantly, 5-HT2A receptor activation triggers retrograde endocannabinoid signaling to reduce GABA release selectively at synapses formed by somatostatin (SST+)- but not parvalbumin (PV+)-positive GABAergic interneurons. Altogether, our results highlight the diverse molecular and cell-type-specific mechanisms by which 5-HT signaling modulates inhibitory circuits to shape cortical function.

## Introduction

Serotonin (5-hydroxytryptamine; 5-HT), by regulating neuronal function throughout the brain, exerts an essential influence on cognitive processes, and its dysregulation has been implicated in the etiology of several mental disorders [1]. 5-HT-containing neurons are predominantly located in the dorsal subdivision of the raphe nucleus (dorsal raphe nucleus (DRN)), from which they project to many brain regions, including the prefrontal cortex (PFC) [2–4]. There, 5-HT-mediated regulation of neuronal function occurs through different 5-HT receptors (5-HTRs), including 5-HT1ARs that reduce the firing and activity of expressing neurons, and 5-HT2A/CRs that mediate excitatory actions on expressing neurons. In glutamatergic neurons, the 5-HT1AR is mainly expressed in the basal dendrites and the axon hillock [5, 6], while the 5-HT2AR is expressed in apical dendrites and thalamocortical terminals in layer 1 [7–9], where it reportedly regulates excitatory synaptic function [7, 10–12]. 5-HTRs are also located in GABAergic interneurons (INs) [1, 13–16], but much less is known about the role of 5-HT receptors in regulating inhibitory synaptic function.

5-HT1Rs and 5-HT2Rs are G-protein coupled receptors that lead to different intracellular signaling cascades activation to regulate neuronal excitability and synaptic function. While 5-HT1Rs are primarily coupled to G_i_/G_o_ proteins to reduce cAMP levels and synaptic transmission, 5-HT2Rs are Gαq_/11_-coupled receptors that, by increasing IP3 and Ca^2+^ levels, generally lead to excitatory effects and enhance synaptic function [17]. Interestingly, like other Gαq_/11_-coupled receptors [18–20], when expressed in postsynaptic neurons, 5-HT2Rs can also mobilize endocannabinoids (eCBs) to suppress synaptic function [18, 21]. However, it is unclear whether 5-HT2ARs up- or down-regulate inhibitory synaptic transmission and plasticity in the mPFC.

Here, using optogenetics and electrophysiological recordings in acute mPFC slices, we evaluated the role of 5-HT in regulating GABAergic synaptic function. We found that both exogenous delivery of 5-HT1AR and 5-HT2AR agonists, as well as the endogenous release of 5-HT, induce a long-lasting suppression of GABA release onto layer 2/3 PNs in the mPFC. Moreover, we found that 5-HT2AR-mediated depression of GABA release depends on the type 1 cannabinoid receptor (CB1R) and is selectively expressed at synapses made by somatostatin (SST)- but not parvalbumin (PV)-positive GABAergic INs. These results identify different forms of 5-HT-mediated regulation of GABAergic synaptic strength at central synapses, highlighting a functional crosstalk between 5-HT2AR and CB1R to regulate GABA release in mPFC.

## Materials and methods

### Animals

Male and female wild-type (C57BL/6), SST-cre (Jackson Laboratory Strain #:013044), PV-cre (Jackson Laboratory Strain #:017320), Fev-cre (Jackson Laboratory Strain #:012712), and conditional SST-CB1 KO mice [22, 23], postnatal days 21–30 were used. Mice were maintained at 18–20 °C on a 12-h light/12-h dark cycle with food and water ad-libitum. Animal handling and use followed a protocol approved by the bioethics committee of the Universidad de Valparaiso (BEA159-20) in accordance with the bioethics regulation of the Chilean Agencia Nacional de Investigación y Desarrollo (ANID).

### Electrophysiological and optogenetics procedures

**Fig. 1:**
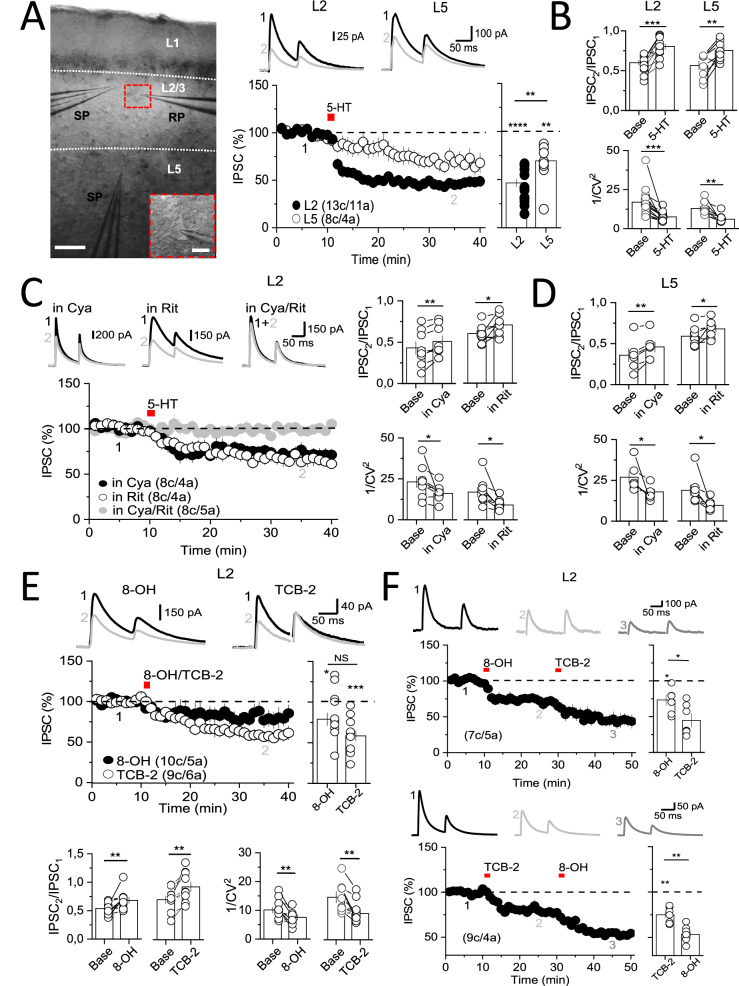
Serotonin presynaptically reduced GABAergic inhibitory inputs onto layer 2/3 pyramidal neurons of the medial prefrontal cortex. **A** *Left:* Representative image showing the localization of the recording (RP) and stimulating (SP) pipettes in layer 2/3 and layer 5 of mPFC. Insets show the recording pipette in one pyramidal neuron that was identified by its shape and visualized using an infrared differential interference contrast video micrograph (IR-DIC). Scale bar: 200 μm (high magnification image) and 25 μm (inset image). *Right*: Representative traces and summarized plots showing that bath application of 5-HT (2 min, 50 µM) depresses eIPSCs elicited by electrically stimulating GABAergic inputs in layer 2/3 or layer 5. **B** 5-HT-induced depression of eIPSCs was accompanied by changes in paired-pulse ratio (PPR; *top panel*) and the coefficient of variation (1/CV^2^; *bottom panel*). **C** Representative traces and summarized plots showing that 5-HT-mediated depression in layer 2/3 was barely reduced by either 5-HT1R antagonist cyanopindolol (Cya, 5 µM; *black dots*) or 5-HT2R antagonist ritanserin (Rit, 4 µM; *open dots*) but was reduced when both antagonists were applied together (gray dots). Changes in PPR and 1/CV^2^ were observed in the presence of individual antagonists. **D** Similar changes in PPR and 1/CV^2^ in the presence of Cya or Rit were observed in layer 5. **E** Representative traces and summarized plots showing that activation of 5-HT1ARs (*black dots*) or 5-HT2ARs (*open dots*) with 8-OH DPAT (8-OH; 3 µM) and TCB-2 (3 µM), respectively, depressed eIPSCs in L2/3, an effect that was accompanied by changes in PPR and 1/CV^2^. **F** Representative traces and summarized plots showing that bath application of 8-OH depresses eIPSCs and subsequent application of TCB-2 further reduced eIPSC. Similarly, TCB-2-mediated depression does not occlude 8-OH-mediated depression. In all panels, data are presented as mean ± SEM, and average sample traces taken at times indicated by numbers are shown next to each summary plot. The number of cells (c) and animals (a) are indicated in parentheses. *P < 0.05, **P < 0.01, ***P < 0.001, ****P < 0.0001. NS not significant.

**Fig. 2:**
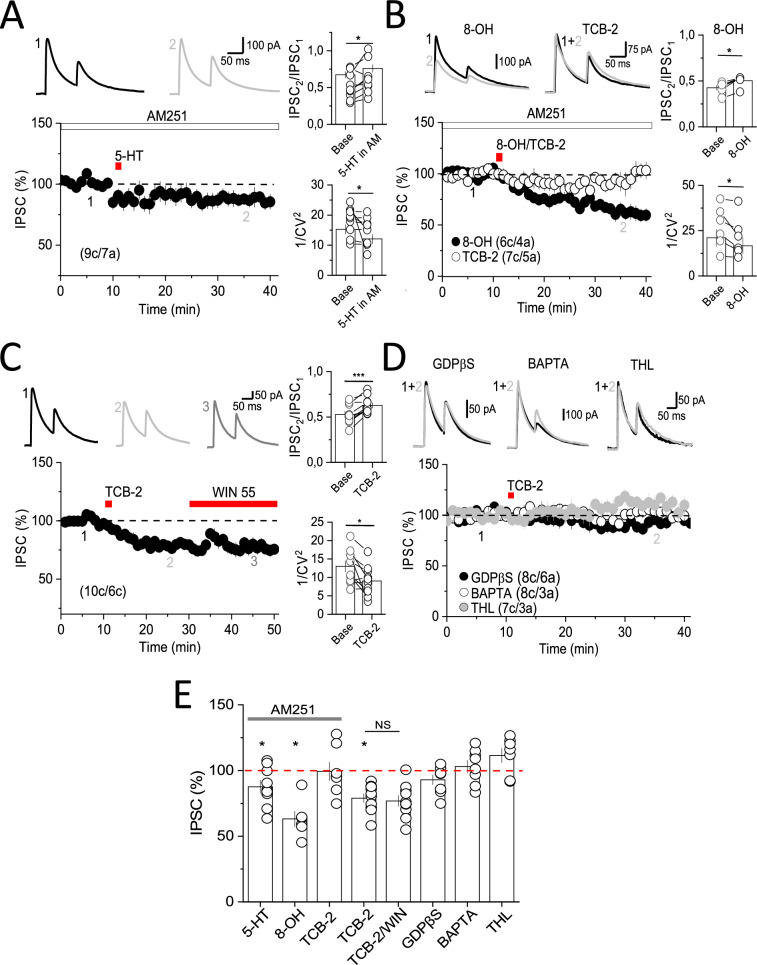
CB1 receptors are required for 5-HT2AR-induced depression of GABA release. **A** 5-HT-mediated depression of eIPSCs was reduced but not completely eliminated in the presence of the CB1R inverse agonist AM251 (4 µM). The remaining depression was accompanied by changes in PPR and 1/CV^2^. **B** Blocking CB1Rs eliminated TCB-2-induced depression of eIPSC, whereas 8-OH DPAT-induced depression of eIPSC was unaffected and was accompanied by changes in PPR and 1/CV^2^. **C** CB1R-mediated depression was occluded by previous activation of 5-HT2AR with TCB-2. **D** TCB-2-mediated depression of eIPSCs was eliminated when PNs were loaded with GDPβS (2 mM), the Ca^2+^ chelator BAPTA (20 mM), or the endocannabinoid 2-AG production blocker THL (4 µM). **E** Summary plot showing the effect of all pharmacological manipulations with respect to basal inhibitory transmission. In all panels, data are presented as mean ± SEM, and representative traces taken at times indicated by numbers are shown. The number of cells (c) and animals (a) are indicated in parentheses. *P < 0.05, ***P < 0.001. NS not significant.

Acute coronal brain slices (300 μm thick) were prepared as previously described [24]. Briefly, the brain was removed following isoflurane anesthesia and cut using a DKT or Leica VT1200S Vibratome in an ice-cold high-choline artificial cerebral spinal fluid (ACSF) solution containing (in mM): 110 Choline-Cl, 2.5 KCl. 1.25 NaH_2_PO_4_, 7 MgCl_2_, 25 NaHCO_3_, 15 glucose, 0.5 CaCl_2_, 11.6 ascorbate, 3.1 pyruvate, pH 7.4 (290–305 mmol/Kg) and equilibrated with 95% O_2_ and 5% CO_2_. Brain slices were kept at room temperature for at least 30 min before recording in ACSF solution containing (in mM): 124 NaCl, 2.69 KCl. 1.25 KH_2_PO_4_, 1.3 MgSO_4_, 26 NaHCO_3_, 10 glucose, 2.5 CaCl_2_, pH 7.4 (300–305 mmol/Kg). Slices containing the mPFC were visualized using infrared differential interference contrast (DIC) video on a fixed-stage Nikon FN1 microscope. Layer 2/3 pyramidal neurons (PNs) from the prelimbic (PrL) subdivision of mPFC were identified by their soma shape and localization (Fig. 1A). Whole-cell voltage-clamp recordings were performed from PNs somas (~200 μm from pia) voltage clamped at 0 mV, using a Multiclamp 700B amplifier (Molecular Devices, Sunnyvale, CA, USA) and patch-type pipette electrodes (~3.0–4.5 MΩ) filled with intracellular solution containing (in mM): 131 Cs-gluconate, 8 NaCl, 1 CaCl2, 10 EGTA, 10 glucose, 10 HEPES, 5 MgATP, and 0.4 Na_3_GTP; pH 7.2–7.4 (285 mmol/kg). In some experiments (Fig. 2), guanosine 5′-[β-thio] diphosphate (GDPβS, 2 mM), the α-diacylglycerol lipase (αDGL) inhibitor THL (4 µM), or BAPTA (20 mM) were included in the intracellular solution as previously described [25].

All experiments were performed at controlled temperature (28 ± 2 °C) in a submersion-type recording chamber perfused at ~1 mL/min supplemented with 40 μM CNQX and 50 μM D-AP-V to block AMPA/kainate and NMDA receptors, respectively. Postsynaptic inhibitory GABAergic currents (IPSC) were elicited by stimulating GABAergic inputs (10–1000 µA) with a bipolar-stimulating patch pipette filled with ACSF and placed in layer 2/3 (100–150 μm from recording cell) or by selective optogenetics activation of SST^+^ or PV^+^ GABA-INs (two pulses at 100 ms inter-stimulus interval (ISI), 1 ms duration). To control GABA release from these GABA-INs, 3-week-old SST- or PV-cre mice were anesthetized (2% isofluorane), placed in a stereotaxic frame (Kopf instruments) and injected into the mPFC with Channelrhodopsin (ChR2)-EYFP fusion protein under the Ef1a promoter (rAAV5 EF1a-DIO-hChR2(H134R)-eYFP; UNC Vector Core, Chapel Hill, North Carolina) following coordinates concerning bregma in mm: 2.1 AP; ±0.5 ML; 1.5 DV. A volume of 0.8–1 µl/site of the virus was injected at a rate of 0.1 µl/min. Postsynaptic GABA response was also elicited by directly puffing GABA (1 mM, 25 ms, 2–4 psi) using a Picospritzer III (Parker Instruments) connected to a patch pipette (~3–4 MΩ). The tip of the puffer pipette was positioned ~150 µm away from the recorded cell in the border between layers 2/3 and 5 and ~30 µm deep from the surface of the slice. To elicit endogenous release of 5-HT in the mPFC, 3-week-old Fev-cre mice were injected into the DRN with the same ChR2-EYFP fusion protein under the Ef1a promoter (but serotype AAV9). DRN was targeted to bregma in mm: 4.6 AP; ± 1.9 ML; 3.5 DV, at a 30° angle. In some experiments (Supplementary Fig. 6), DRN neurons were recorded in current clamp mode using an intracellular solution containing (in mM): 135 KMeSO3, 10 HEPES, 4 MgCl2, 4 Na2ATP, 0.4 NaGTP, and 10 sodium creatine phosphate, adjusted to pH 7.3 with KOH. Injected SST- and PV-cre mice were used for electrophysiological procedures 2 weeks after ChR2 injection, while Fev-cre mice were used 3 weeks after ChR2 injection.

GABAergic IPSC paired-pulse ratio (PPR, 100 ms ISI) was defined as the ratio of the amplitude of the second IPSC to the amplitude of the first IPSC. The coefficient of variation (1/CV^2^) was calculated as the squared mean IPSC amplitude divided by IPSC variance. Both PPR and 1/CV^2^ were calculated 10 min before and 15–20 min after the pharmacological activation of 5-HTRs or 5-HT-mediated long-term depression (LTD) induction protocol (a train of 5 light pulses (20 ms inter-stimulus interval; 50 Hz) every 6 s delivered 30 times at 0.17 Hz; Supplementary Fig. 6A, E). Pharmacological agents were bath applied after the establishment of a stable baseline (~10–15 min), and their effects were measured after responses reached a new steady state (typically >15 min). Drugs were obtained from Sigma and Tocris, prepared in stock solutions in water or DMSO, and added to the ACSF as needed. Total DMSO in the ACSF was maintained at <0.01%.

GABAergic IPSCs were elicited at 15-sec intervals (0.25 Hz), except for exogenous GABA puffs that were elicited at 30-sec intervals (0.5 Hz), filtered at 2.2 kHz, and acquired at 10 kHz using custom-made software written in Igor Pro 4.09A (WaveMetrics). Series resistance ( ~ 10–25 MΩ) was monitored throughout all experiments with a −4 mV, 80 ms voltage step, and cells that exhibited a significant change in series resistance (>20%) were excluded from the analysis.

Unless otherwise indicated, statistical analysis and graphical data formatting were performed using GraphPad Prism 8 or Origin Pro 8 software. All data distributions were tested for normality using the Shapiro-Wilk test. Normal data distributions underwent parametric paired or unpaired *t*-test analysis, whereas for skewed distributions or small sample sizes, the nonparametric paired two-tailed Student *t*-test (Wilcoxon matched-pairs signed rank test) was applied. When three or more groups of data were compared, one-way (for one independent variable) or two-way (for two independent variables) ANOVA was performed. In contrast, for a nonparametric distribution of the data, the Friedman test was used. Before ANOVA, a multivariate normality test (Mauchly’s test of sphericity) was used to assess data homoscedasticity. To reveal differences among groups, *post-hoc* parametric Tukey´s or nonparametric Dunn multiple comparison tests were conducted, while for comparing several data sets to a single data group, Dunnett’s multiple comparison test was used. Statistical significance was determined for *p* values < 0.05 associated with the statistical analysis test applied. Values are provided as the mean ± SEM, and figure traces are averages of 25–30 responses, except for the GABA puff response, where 5–10 responses were used.

### Immunohistochemistry

SST-, PV-, and Fev-cre mice intracranially injected with ChR2-EYFP were fixed through transcardial perfusion with 4% paraformaldehyde, the brains were removed and post-fixed with 4% paraformaldehyde in 0.1 M phosphate buffer (PBS; pH 7.4) for 2 h and maintained in 30% sucrose for at least 48 h. Brain sections (30 µm thick) were cut on a freezing-stage cryostat (Leica CM1850), blocked with 3% normal horse serum in PBS, and incubated with chicken anti-green fluorescent protein (1/1000; Invitrogen, Thermofischer #A10262) and anti-tryptophan hydroxylase (TPH; 1/1000; Abcam, #ab111828) primary antibodies overnight at room temperature. Then, sections were incubated in goat anti-chicken Alexa Fluor 488 (Invitrogen, Thermofischer #A-11039) and Alexa Fluor 594-conjugated donkey anti-rabbit (1/1000; Jackson ImmunoResearch #111-585-045) secondary antibodies. Three 10-min washes were performed in between and after incubation in antibody solutions. Finally, sections were mounted on glass slides and coverslipped with Vectashield containing DAPI antifade reagent (Vector Laboratories). Images were acquired in a Nikon C1 Plus confocal microscope and analyzed using the ImageJ software as previously described [26].

## Results

### Brief 5-HT exposure reduces the strength of GABAergic inputs in the medial prefrontal cortex

The effects of 5-HT on GABAergic synaptic transmission onto layer 2/3 pyramidal neurons of the mPFC were examined by measuring inhibitory postsynaptic currents (eIPSCs) elicited by electrically stimulating GABAergic inputs in layer 2/3 or layer 5 (paired-pulse, 100 ms ISI; Fig. 1A). Transient application of 5-HT (2 min, 50 µM) significantly reduced eIPSCs evoked by stimulation of GABAergic inputs in layer 2/3 as well as those stimulated in layer 5 (Fig. 1A, Supplementary Table 1). This depression of eIPSCs was associated with changes in the PPR and the coefficient of variation (1/CV^2^; Fig. 1B, Supplementary Table 1), two independent measurements that suggest a presynaptic locus of plasticity expression. Accordingly, 5-HT also reduced the frequency of spontaneous GABAergic IPSCs (sIPSCs) without affecting their amplitude (Supplementary Fig. 1A, Supplementary Table 2) and had no effect on GABA-induced currents elicited by brief puffs of GABA (1 mM), a manipulation known to short-cut transmitter release (Supplementary Fig. 1B, Supplementary Table 2). Altogether, these results indicate that 5-HT-mediated depression of synaptic inhibition is presynaptically expressed. We next investigated the cellular mechanism underlying this 5-HT-mediated depression.

First, we aimed to identify the 5-HT receptor subtype(s) involved in this decrease of GABA release. 5-HT-induced depression of eIPSC was barely reduced by bath application of either cyanopindolol (Cya, 5 μM), a 5-HT1R antagonist, or by ritanserin (Rit, 4 μM), a 5-HT2R antagonist, but was eliminated when both antagonists were applied together in both layer 2/3 (Fig. 1C; Supplementary Table 1) and layer 5 (Supplementary Fig. 2A; Supplementary Table 1). Importantly, 5-HT-mediated depression in the presence of Cya or Rit was associated with changes in PPR and 1/CV^2^ in both layer 2/3 (Fig. 1C) and layer 5 (Fig. 1D; Supplementary Fig. 2A). Likewise, two other 5-HT1R (WAY100635, 1 µM) and 5-HT2R (MDL100907, 0.2 µM) antagonists also reduced 5-HT-mediated depression in L2/3 synapses when applied together but not when applied alone (Supplementary Fig. 2B; Supplementary Table 1). As equivalent results were observed between layer 2/3 and layer 5 inputs (Supplementary Fig. 2C), our subsequent focus was on GABAergic inputs in layer 2/3 to evaluate the cellular mechanism by which 5-HT1Rs and 5-HT2Rs induce this presynaptic depression of inhibitory inputs in the mPFC.

Second, we aimed to further dissect the contribution of specific 5-HT1Rs and 5-HT2Rs subtypes on eIPSCs using more selective agonists. Bath application of CP-93129 (3 μM), a specific 5-HT1BR agonist, had no effect on eIPSCs recorded in layer 2/3 and layer 5 of the mPFC (Supplementary Fig. 3A). As a positive control, in naïve slices, application of CP-93129 (3 μM) was able to reduce the field excitatory postsynaptic potential (fEPSP) recorded in the perforant path (PP) input to CA1 hippocampal region. Importantly, CP-93129-mediated depression of fEPSP was accompanied by changes in both PPR and 1/CV^2^ and was eliminated by the specific 5-HT1B antagonist NAS-181 (6 µM; Supplementary Fig. 3B and Supplementary Table 2). However, application of NAS-181 (6 µM) had no effect on 5-HT-induced depression of eIPSC (Supplementary Fig. 3C), suggesting that 5-HT1BR are not involved in the depression of eIPSC in the mPFC. In contrast, bath application of 8-OH DPAT (8-OH, 3 µM), a 5-HT1AR agonist, induced a long-lasting reduction of eIPSC amplitude that was associated with changes in PPR and 1/CV^2^ (Fig. 1E, Supplementary Table 1). Similarly, bath application of TCB-2 (3 µM), a specific 5-HT2AR agonist, but not WAY 161503 (3 µM), a specific 5-HT2CR agonist (Supplementary Fig. 3D), also reduced eIPSCs in a long-lasting manner with changes in both PPR and 1/CV^2^ (Fig. 1E, Supplementary Table 1). Importantly, TCB-2-mediated depression was also normally induced on synapses that already expressed 8-OH DPAT-mediated depression (Fig. 1F). Inversely, 8-OH DPAT-mediated depression was still able to suppress eIPSC, having already expressed TCB-2-mediated depression (Fig. 1F). These experiments indicate that 5-HT1AR- and 5-HT2AR-mediated synaptic depression do not share a common mechanistic step to induce presynaptic changes in GABA release.

Third, we reasoned that if 5-HT1AR- and 5-HT2ARs are located postsynaptically to mediate depression of eIPSC, loading pyramidal cells (PNs) with the G protein blocker GDPβS (2 mM) should block their effects. While bath application of TCB-2 (3 µM) no longer reduced eIPSC amplitude, subsequent application of 8-OH DPAT (3 µM) still markedly reduced eIPSCs, an effect that was associated with changes in PPR (Supplementary Fig. 4A, Supplementary Table 2). Taken together, these results support the idea that presynaptic 5-HT1AR and postsynaptic 5-HT2AR are involved in the reduction of GABA release by 5-HT. This interpretation is further bolstered by the observation that TCB-2 does not suppress GABA-induced current elicited by a brief puff of GABA (Supplementary Fig. 4B; Supplementary Table 2). Altogether, these results indicate that both presynaptic 5-HT1ARs and postsynaptic 5-HT2ARs can act to reduce GABA release onto layer 2/3 PNs.

### Cannabinoid signaling is required for 5-HT2R-mediated presynaptic depression

How could postsynaptic 5-HT2ARs reduce presynaptic GABA release? Like other Gαq_/11_-coupled receptors, the 5-HT2AR is coupled to phospholipase C (PLC) and diacylglycerol lipase (DGL) that may engage eCB production and subsequent activation of presynaptic type 1 cannabinoid receptors (CB1Rs) to reportedly depress excitatory synaptic transmission in the inferior olive and nucleus accumbens [18, 21]. To test this possibility at GABAergic synapses in the mPFC, first, we confirmed the involvement of CB1Rs in the reduction of GABAergic IPSC in the mPFC by applying the CB1R agonist WIN 55,212 (WIN, 5 µM) [27]. Accordingly, WIN markedly reduced eIPSC amplitude, an effect that has been associated with changes in PPR and 1/CV^2^ and was eliminated in slices previously incubated with the CB1R inverse agonist AM251 (4 µM; Supplementary Fig. 5, Supplementary Table 2). Next, we evaluated whether 5-HT-mediated depression requires activation of CB1Rs. To this end, we applied 5-HT in slices pre-incubated with AM251 and found that 5-HT-mediated depression was significantly reduced, and the remaining depression was associated with changes in PPR and 1/CV^2^ (Fig. 2A, E; Supplementary Table 1). Importantly, the suppression of eIPSC induced by the specific activation of 5-HT2ARs with TCB-2 was eliminated in slices pre-incubated with AM251 (Fig. 2B, E), whereas 8-OH DPAT reduced IPSC (Fig. 2B, E; Supplementary Table 1), further bolstering our previous observation that two different receptors and mechanisms (5-HT1AR and 5-HT2AR) are involved in the 5-HT-mediated depression of eIPSC. Remarkably, the activation of CB1R after TCB-2-induced depression did not induce further effects (Fig. 2C, E), suggesting that 5-HT2ARs and CB1Rs share a common mechanism of action to depress GABA release. To test this idea, next we loaded PNs with the G-protein blocker GDPβs (2 mM), and under this condition, TCB-2 no longer reduced eIPSCs (Fig. 2D, E, Supplementary Table 1). Likewise, interfering with the production of the eCB 2-arachidonoylglycerol (2-AG) by loading PNs with the Ca^2+^ chelator BAPTA (20 mM) or with the diacylglycerol lipase (αDGL) inhibitor tetrahydrolipstatin (THL, 4 µM; Fig. 2D, E, Supplementary Table 1) also eliminated TCB-2-induced suppression of eIPSCs. Altogether, these results indicate that postsynaptic 5-HT2AR inhibits GABAergic IPSCs by releasing eCB to act in presynaptic CB1R located in GABAergic terminals, further supporting a relationship between 5-HT2AR activation and CB1R to regulate inhibitory afferents onto PNs of the mPFC.

### 5-HT2AR-mediated GABAergic LTD is synapse-type specific

**Fig. 3:**
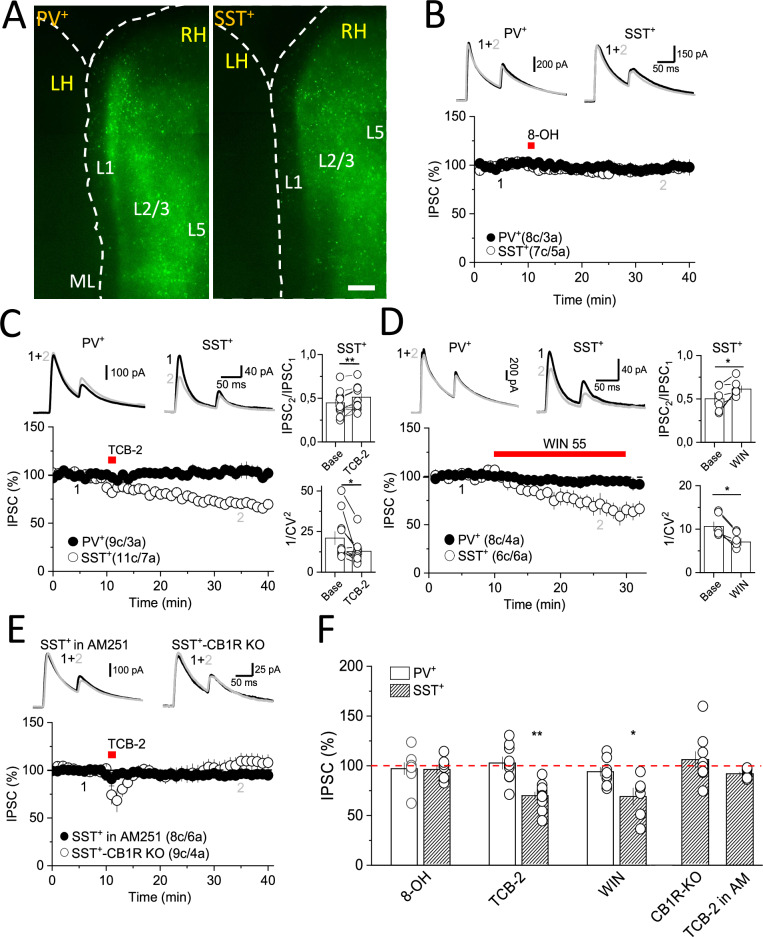
GABA release from SST^+^ interneurons is selectively depressed by 5-HT2AR and CB1R activation. **A** Fluorescent confocal images showing conditional expression of ChR2-YFP construct in PV^+^- (*Left*) and SST^+^-INs (*Right*) in the mPFC. Viral infection was performed only in the right hemisphere (RH), while the left hemisphere (LH) was not infected (used as a positive control). ML: midline, L1: layer 1, L2/3: layer 2/ 3, and L5: layer 5. Scale bar: 200 μm. **B** Representative traces (*top*) and summarized plot (*bottom*) showing that optogenetic activation of GABA release from PV^+-^ (PV^+^-IPSC; *black dots*) or SST^+^-IPSC (*open dots*) was unaffected by bath application of 5-HT1AR agonist 8-OH DPAT (8-OH, 3 µM). **C** Activation of 5-HT2AR with TCB-2 (3 µM) significantly reduces SST^+^-IPSCs (*open dots*) but have no effect on PV^+^-IPSCs (*black dots*). Note that TCB-2-induced depression was associated with changes in PPR and 1/CV^2^. **D** SST^+^-IPSCs (*open dots*) but not PV^+^-IPSCs (*black dots*) were also reduced by bath application of the CB1R agonist WIN 55,212 (WIN, 5 µM), an effect that was accompanied by changes in PPR and 1/CV^2^. **E** TCB-2-induced depression of SST^+^-IPSCs was eliminated in slices pre-incubated with the CB1R inverse agonist AM251 (4 µM; *black dots*) and in slices from selective SST^+^-CB1R knock-out cells (SST^+^-CB1R KO, *open dots*). **F** Summary plot showing the effect of all pharmacological manipulations with respect to basal inhibitory from PV^+^ (*open bar*) or SST^+^ (*dashed bar*). In all panels, data are presented as mean ± SEM, and the number of cells (c) and animals (a) are indicated in parentheses. *P < 0.05, **P < 0.01.

Different GABAergic interneurons are expressed in the mPFC, with somatostatin- (SST^+^) and parvalbumin-positive (PV^+^) subtypes being the more abundant populations [28]. To evaluate the ability of 5-HT1AR and 5-HT2AR to regulate specific GABAergic synapses, we took advantage of cell-type-specific optogenetic stimulation by expressing ChR2-EYFP on PV^+^- and SST^+^-INs (see “Methods” and Fig. 3A). Paired-pulse light stimulation (100 ms ISI, 1 ms duration) elicited IPSCs mediated by PV^+^-INs (PV-IPSCs) that were unaffected by bath application of 8-OH DPAT (3 µM; Fig. 3B, F). Similarly, bath applications of 8-OH DPAT did not affect IPSC mediated by SST^+^-INs (SST-IPSCs) (Fig. 3B, F), suggesting that other GABAergic INs subtypes, rather than PV^+^ or SST + -INs synapse, are modulated by 5-HT1AR in the mPFC. In contrast, we found that bath application of 5-HT2AR agonist TCB-2 (3 µM; Fig. 3C, Supplementary Table 1) had no effect on PV-IPSCs but significantly reduced SST-IPSCs (Fig. 3C, F, Supplementary Table 1). Consistent with a presynaptic mechanism of action of TCB-2, the reduction of SST-IPSC was accompanied by changes in PPR and 1/CV^2^ (Fig. 3C, Supplementary Table 1). To further determine whether activation of CB1R is required for the 5-HT2AR-mediated depression of SST^+^ synapses, first, we bath applied the CB1R agonist WIN (5 μM) while photostimulating SST^+^-INs and found that WIN significantly reduced SST-IPSCs with changes in both PPR and 1/CV^2^, but had no effect on PV-IPSCs (Fig. 3D, Supplementary Table 1). More importantly, 5-HT2AR-mediated suppression of SST-IPSCs was eliminated in the continuous presence of the CB1R inverse agonist AM251 (Fig. 3E, F) and was absent when CB1Rs were selectively deleted from SST^+^-INs (SST-CB1R KO) [22, 23] (Fig. 3E, F). Altogether, these results indicate that 5-HT2AR activation induces a synapse-specific depression of GABA release that requires CB1R activation at SST^+^-, but not PV^+^- synapses in the mPFC.

### Endogenous 5-HT also reduces GABA release in a 5-HT2AR and CB1R-dependent manner

**Fig. 4:**
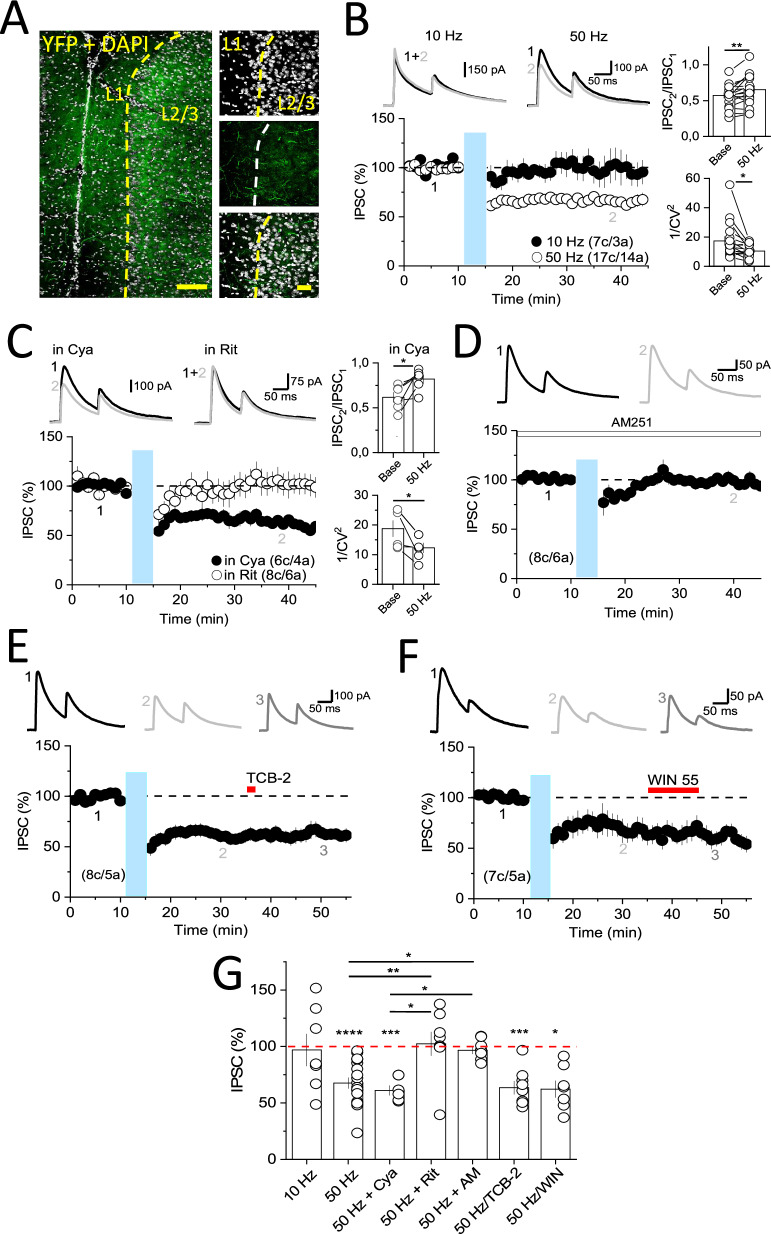
Repetitive optogenetic activation of serotonergic fibers in the mPFC induced LTD of GABAergic IPSC in a 5-HT2A and CB1R-dependent manner. **A** Confocal images of DRN neuron terminals in mPFC (YFP; *green label*) expressing ChR2. DAPI (*gray label*) labels the cellular nucleus in mPFC. Scale bar: 200 and 50 μm for low and high magnification images, respectively. L1: layer 1, L2/3: layer 2/ 3 and L5: layer 5. **B** Representative traces (*top*) and summary plots (*bottom*) showing that 50 Hz but not 10 Hz optogenetic stimulation protocol (*light blue bar*) triggers a long-lasting depression (LTD) of eIPSCs in layer 2/3 of mPFC. 50-Hz-induced LTD was accompanied by changes in PPR and 1/CV^2^. **C** Representative traces and summary plots showing that 50 Hz light stimulation was unaffected by the 5-HT1R antagonist cyanopindolol (Cya, 5 µM; *black dots*) but eliminated in the presence of the 5-HT2R antagonist ritanserin (Rit, 4 µM; *open dots*). Note that 50-Hz-induced LTD in the presence of cyanopindolol was accompanied by changes in PPR and 1/CV^2^. **D** 50-Hz-induced LTD was also eliminated in slices pre-incubated with the CB1R inverse agonist AM251 (4 µM). **E** Representative traces and summary plots showing that bath application of TCB-2 (3 μM) has no effect on eIPSCs already depressed by the 50 Hz stimulation protocol. **F** Similarly, activation of CB1R with WIN 55,212 (WIN, 5 µM) has no effect on eIPSCs already depressed by a 50 Hz stimulation protocol. **G** Summary plot showing the effect of 10 Hz and 50 Hz induction protocol on GABAergic IPSCs in the presence of different pharmacological agents. In all panels, data are presented as mean ± SEM, and average sample traces taken at times indicated by numbers are shown next to each summary plot. The number of cells (c) and animals (a) are indicated in parentheses. *P < 0.05, **P < 0.01, ***P < 0.001, ****P < 0.0001.

To further evaluate whether the endogenous release of 5-HT in the mPFC could also trigger a long-lasting depression of GABA release, we took advantage of Fev-cre mice to express ChR2-EYFP specifically in serotonergic neurons within the DRN and optogenetically activated their terminals in layer 2/3 of the mPFC (Supplementary Fig. 6A). Importantly, immunohistochemistry sections of DRN revealed colocalization of ChR2-EYFP with TPH label (Supplementary Fig. 6B), and light pulses (50 ms) induced an inward current and depolarization of DRN neurons (Supplementary Fig. 6C). Moreover, immunohistochemistry section of the mPFC confirmed ChR2-EYFP expression in serotonergic DRN terminals that extended along mPFC (Fig. 4A). Brief light activation of 5-HT terminals (50 ms) also depolarized PNs, an effect that was eliminated in the continuous presence of the 5-HT2R antagonist ritanserin (4 µM; Supplementary Fig. 6D; Supplementary Table 2), further supporting the postsynaptic localization of 5-HT2Rs in PN. These observations prompted us to investigate whether repetitive DRN activity induces 5-HT-dependent form of LTD at GABAergic synapses. To this end, we evaluated whether repetitive activation of DRN at 10 Hz or 50 Hz (see “Methods”; Supplementary Fig. 6E; Supplementary Table 2) can regulate GABAergic plasticity in the mPFC. While 10 Hz stimulation frequency had no effect on eIPSCs, the 50 Hz stimulation protocol was able to induce a long-lasting depression (LTD) of GABAergic IPSCs onto layer 2/3 PNs (Fig. 4B, G; Supplementary Table 1). Similar to exogenous 5-HT-mediated depression (Fig. 1), 50 Hz stimulation of DRN terminals triggered inhibitory LTD that was accompanied by changes in PPR and 1/CV^2^ (Fig. 4B). Moreover, this form of LTD requires 5-HT2Rs but not 5-HT1Rs activation, as it was unaffected by the 5-HT1R antagonist cyanopindolol (5 µM) but was eliminated when slices were pre-incubated with the 5-HT2R antagonist ritanserin (4 µM; Fig. 4C, G). These results indicate that 5-HT2R, but not 5-HT1Rs, are involved in this form of 5-HT-mediated LTD induced by optogenetic stimulation of serotonergic terminals in the mPFC. Remarkably, 5-HT-induced LTD was also eliminated in slices pre-incubated with the CB1R inverse agonist AM251 (4 µM; Fig. 4D, G), indicating that 5-HT2AR and CB1R are also required for this form of LTD. Accordingly, pharmacological activation of either 5-HT2AR (Fig. 4E, G) or CB1R (Fig. 4F, G) after the induction of 5-HT-induced LTD had no further effect on IPSC (Supplementary Table 2), suggesting that these receptors share a common mechanism of action to regulate GABA release and indicates that endogenous 5-HT induced depression involves a crosstalk between 5-HT2AR and CB1R receptors to modulate GABA release in the mPFC.

## Discussion

5-HT is known to regulate synaptic function throughout the brain by activating different receptors and signaling pathways. Here, we identify two presynaptic cellular mechanisms involved in the action of 5-HT to regulate GABAergic synaptic function in layer 2/3 of the mPFC, highlighting a functional crosstalk between 5-HT and eCB systems. Such crosstalk is cell-type specific, impacting SST^+^ but not PV^+^ INs afferents terminals. While 5-HT1AR-mediated depression of GABAergic inputs is likely to be independent of SST^+^ or PV^+^ INs subtype, the 5-HT2AR-mediated suppression of GABA release is synapse-specific and requires the activation of postsynaptic 5-HT2AR that triggers eCB release and activation of CB1Rs in presynaptic terminals of SST^+^ INs. While a large fraction of 5-HT1/5-HT2AR-expressing GABAergic INs are PV-positive fast-spiking INs [29, 30], we found that the application of 8-OH DPAT or TCB-2 had no effect on GABA release from this type of interneuron. One possibility is that the expression of this receptor is postsynaptic to synaptic inputs [7] along the axis regulating other processes rather than synaptic release. Likewise, the presence of 5-HT2AR in SST^+^ INs [14] also suggests that these receptors might also be postsynaptic to excitatory and/or inhibitory inputs rather than regulate GABA release onto PNs. Further experiments will be required to clarify the precise location and function of these receptors within specific types of GABAergic INs to regulate mPFC network activity and behavior.

### 5-HT1A and 5-HT2A receptors regulate GABAergic synaptic transmission in mPFC

Different 5-HTRs are present in mPFC and participate in modulating synaptic transmission onto PNs, including 5-HT1, 5-HT2, 5-HT3, and 5-HT7 receptors [1, 7, 11, 31, 32]. Here, we identified that activation of 5-HT1ARs, but not 5-HT1BR, likely expressed in GABAergic terminals, reduces inhibitory inputs elicited by electrical stimulation (Fig. 1), but not inhibitory inputs elicited by specific stimulation of PV^+^- or SST^+^-INs (Fig. 3). Such results suggest that other types of GABAergic interneuron within the mPFC are likely to be under modulation of presynaptic 5-HT1ARs. The 5-HT1AR-mediated inhibition observed here is non-canonical, as this subtype traditionally acts postsynaptically to regulate neuronal firing. Indeed, 5-HT1AR activation with 8-OH DPAT reportedly reduces neuronal firing of GABAergic INs in the mPFC [33]. Our observation that 5-HT1R activation with 8-OH DPAT reduces GABA release (Fig. 1) may, in principle, be caused by the activation of G_i_/G_o_ protein expressed at synaptic terminals. Further experiments using complementary approaches will be required to directly determine the subcellular localization (i.e., dendritic vs synaptic terminal compartments) of 5-HT1Rs within GABAergic interneurons in the mPFC.

Surprisingly, we also revealed a presynaptic modulation of GABA release by postsynaptic 5-HT2ARs (Figs. 1, 2 and 4). These receptor subtypes are known to be expressed in the soma and dendrites of PNs, where they exert changes in PNs excitability and excitatory synaptic transmission [7, 11, 30, 34]. For instance, postsynaptic 5-HT2ARs seem to play a crucial role in depressing fast excitatory transmission by facilitating AMPA receptor internalization [7, 35], while presynaptic 5-HT2ARs potentiate slow NMDA-mediated excitatory transmission by up-regulating NMDAR activity [10]. Unlike previous evidence showing that activation of 5-HT2Rs reduces inhibition through the phosphorylation of GABA_A_Rs in dissociated PNs [36], here we show that pharmacological activation of 5-HT2ARs in acute mPFC slices had no effect on GABA-puff mediated responses in PNs (Supplementary Fig. 3B). Moreover, our results demonstrate that activation of postsynaptic rather than presynaptic 5-HT2ARs significantly reduces GABA release from SST^+^-INs (Fig. 3). Remarkably, this depression of GABA release is likely due to a functional crosstalk between postsynaptic 5-HT2AR and presynaptic CB1Rs (Figs. 2 and 4) similar to those demonstrated at excitatory inputs in other brain areas, including the inferior olive and the nucleus accumbens [18, 21]. Indeed, our results suggest that 5-HT activates 5-HT2ARs to promote the synthesis and release of the eCB 2-AG that recruits CB1Rs on terminals of SST^+^-INs to reduce GABA release (Figs. 2 and 3). While the functional relevance of this crosstalk remains unclear, both 5-HT and eCB systems have been implicated in fear memory [37, 38], and SST^+^-INs seem to play an important role in regulating fear memory [39, 40]. Further experiments will be required to clarify the subtype of GABAergic interneuron expressing 5-HT1AR-mediated depression of GABA release, as well as to determine whether 5-HT2-CB1R crosstalk is also present at excitatory synapses and its functional relevance in regulating mPFC network activity and behavior.

### 5-HT2AR-mediated LTD in the mPFC is cell-type specific

In the neocortex, three principal groups of interneurons coexist: cells expressing the calcium-binding protein parvalbumin (PV^+^-INs), the peptide somatostatin (SST^+^-INs), or the ionotropic 5-HT3A receptor. PV^+^-INs target perisomatic and axon initial segment regions of PNs, 5-HT3A-expressing INs are formed by a heterogeneous group of cells in more superficial layers, and SST^+^-INs target apical dendrites of PNs both in shafts and spines [28, 41]. Our results not only further support the potential expression of functional CB1R in SST^+^ terminals [42] but also reveal a cellular mechanism by which eCBs are generated to retrogradely modulate SST^+^ synaptic strength. We cannot rule out the possibility that other types of GABAergic interneurons could also express this functional crosstalk between 5-HT2Rs and CB1Rs. Indeed, in the neocortex and also the hippocampus, perisomatic-targeting basket cells and those expressing the neuropeptide cholecystokinin (CCK^+^-INs) co-express CB1Rs, which reportedly modulate GABA release as a result of retrograde signaling from target PNs [43–45]. Further experiments will be necessary to elucidate whether CCK^+^-INs or another subtype also express 5-HT2AR-mediated LTD that requires eCB signaling.

### Frequency-dependent optogenetic delivery of 5-HT

Previous evidence demonstrated that intact DRN 5-HT neurons could reach high-frequency firing rates during burst events in behavioral tasks or noradrenergic stimulation [46–48]. In the striatum and mPFC, a 10 Hz optogenetic stimulation of 5-HT terminals was enough to induce a time-dependent LTD on excitatory transmission by activating 5-HT4Rs and 5-HT2cRs, respectively [49, 50]. However, in our experimental conditions, delivery of 10 Hz stimulation had no effect on GABAergic transmission (Fig. 4B), highlighting the different modulatory roles of 5-HT at excitatory and inhibitory synapses. In contrast, we found that 50 Hz stimulation was able to induce and maintain a 5-HT-mediated LTD of GABAergic transmission that is presynaptically expressed and requires 5-HT2AR and CB1R. These differences could be due to differences in the release probability between excitatory and inhibitory synapses, the localization of the DRN synaptic terminals, or even the differential affinity and/or localization of 5-HTRs within different synaptic terminals. Further studies will be required to understand the frequency dependence of 5-HT mediated changes in synaptic strength in the mPFC, as well as its physiological relevance in regulating the brain cortex network in health and disease.

## Supplementary information

Supplementary Figures

Supplementary Tables 1 and 2

## Data Availability

All study data are included in the article and/or supplementary material and are also available from the corresponding author on reasonable request.
