# Supplementary material for: Serotonin-endocannabinoid crosstalk selectively regulates inhibitory GABAergic inputs in the medial prefrontal cortex
Source: Neuropsychopharmacology. 2026 Feb 11;51(8):1474–84. doi: 10.1038/s41386-026-02364-8 (PMC13291222; doi:10.1038/s41386-026-02364-8)
Supplement: Supplementary file 1 — Supplementary Figures [file 41386_2026_2364_MOESM1_ESM.pdf]

Supplementary Figure 1

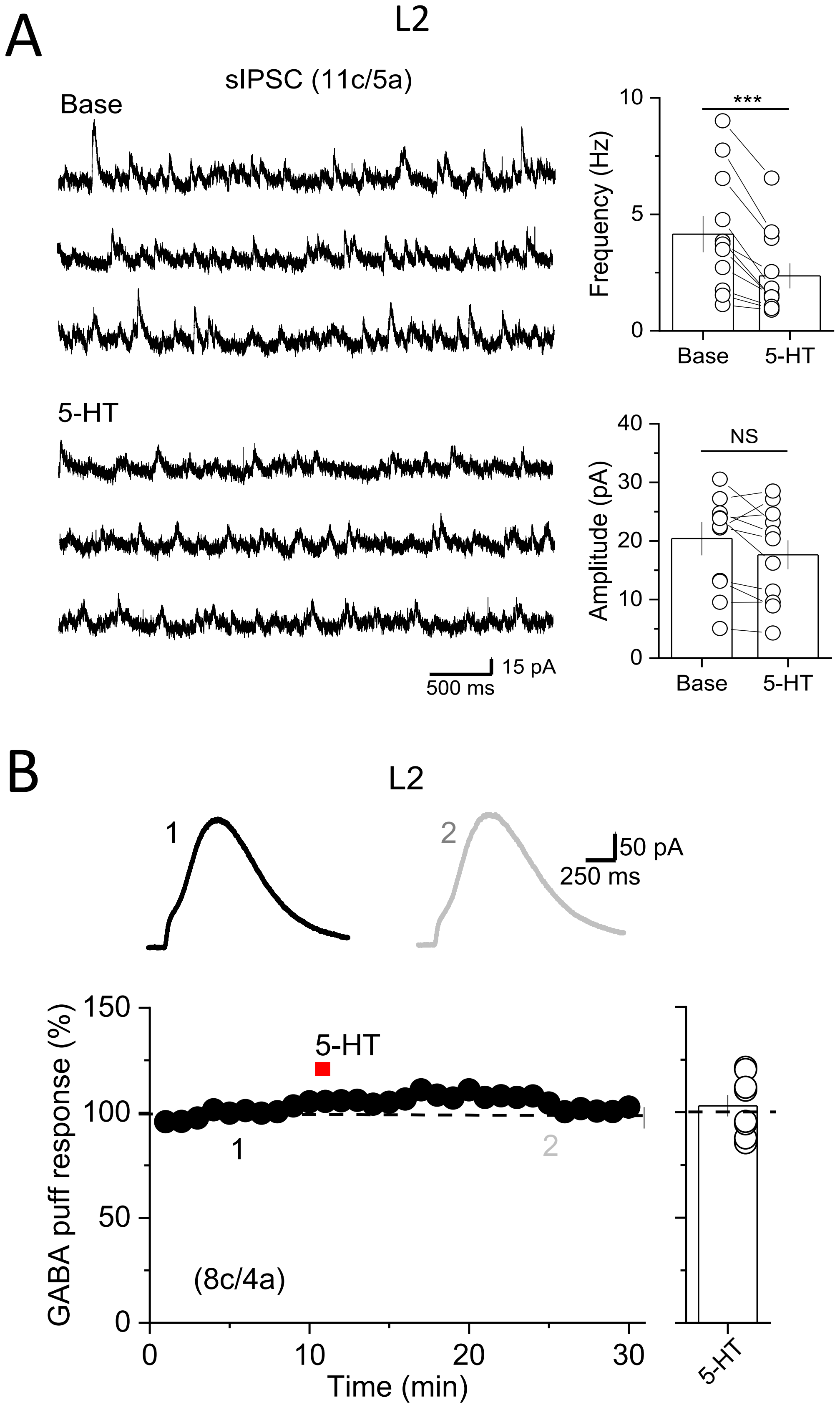

Supplementary Figure 2

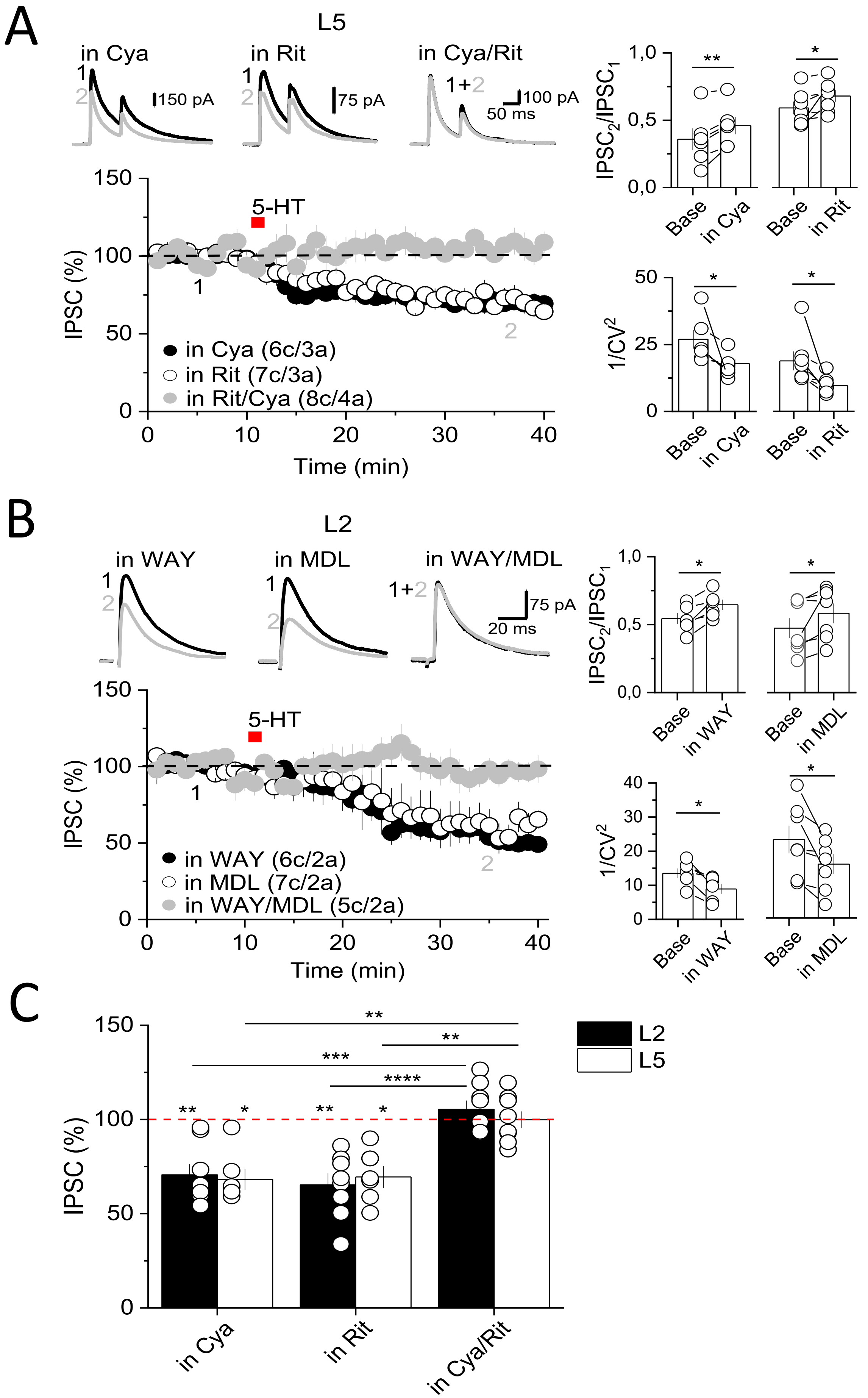

Supplementary Figure 3

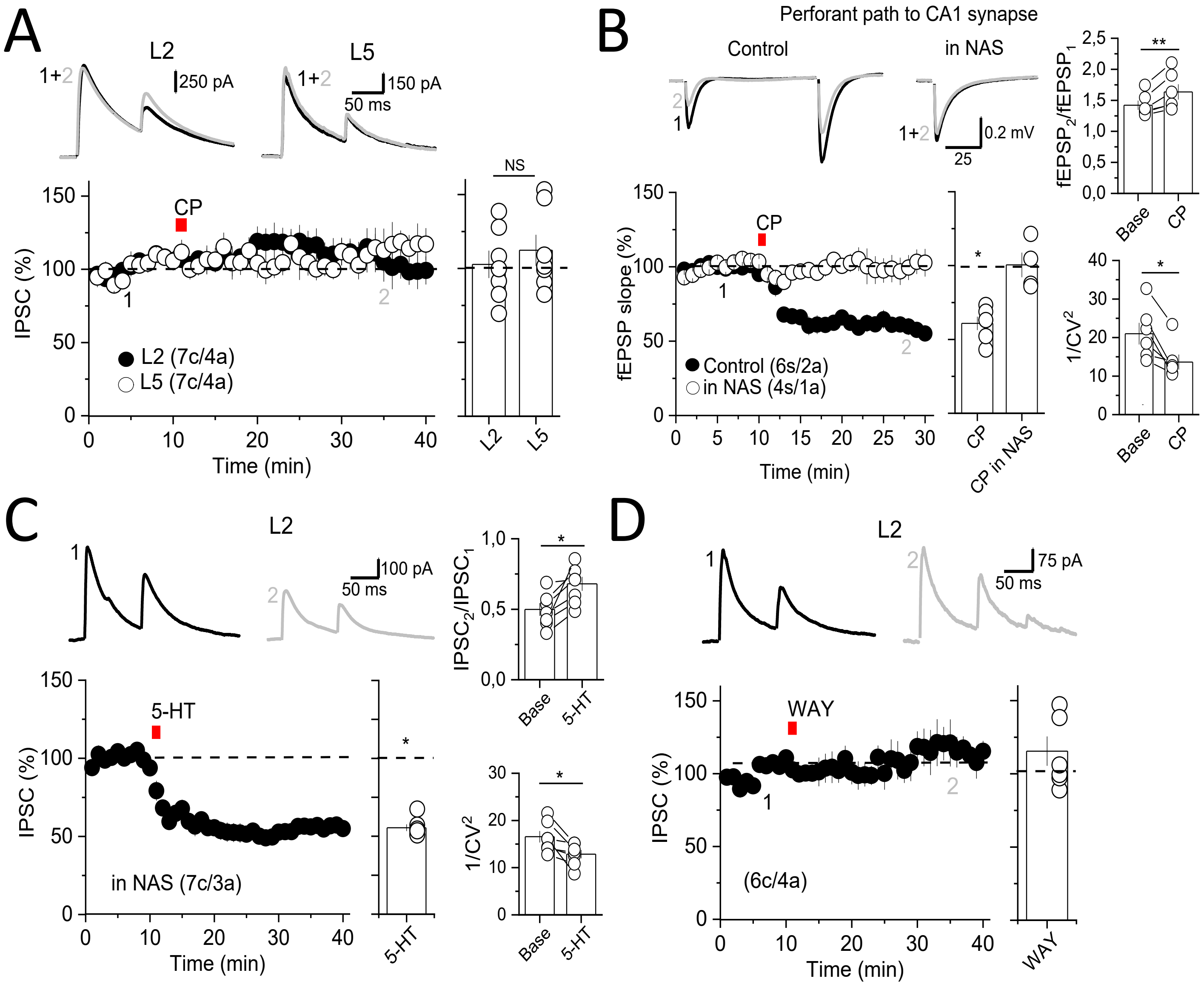

Supplementary Figure 4

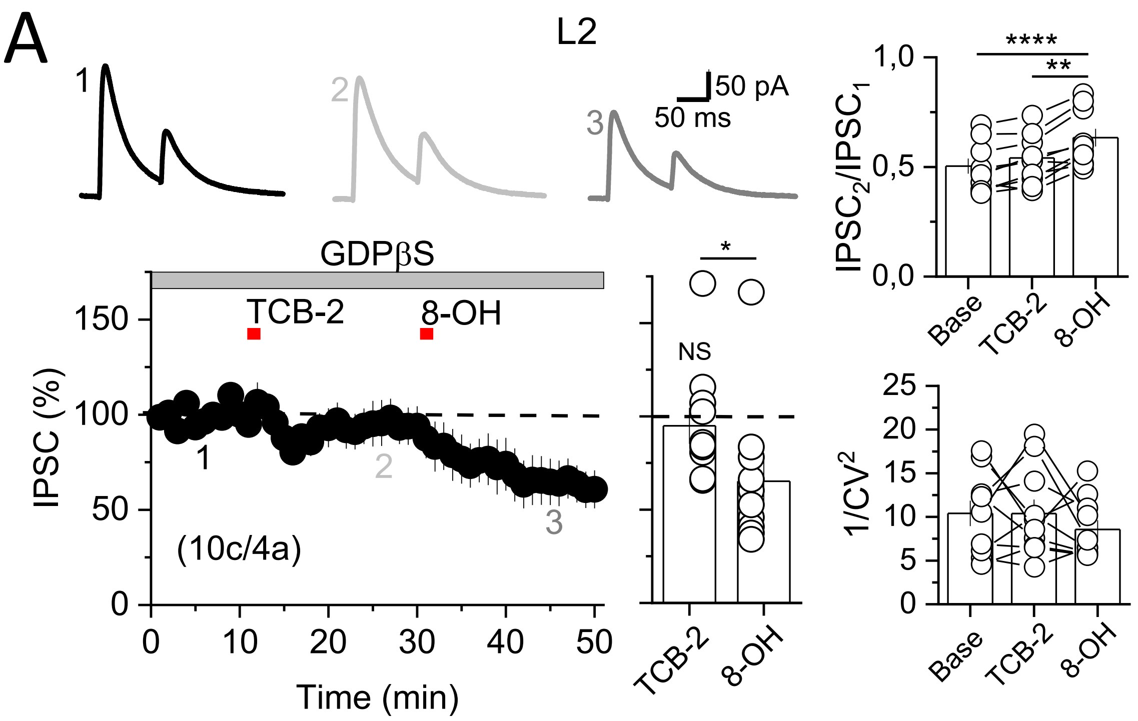

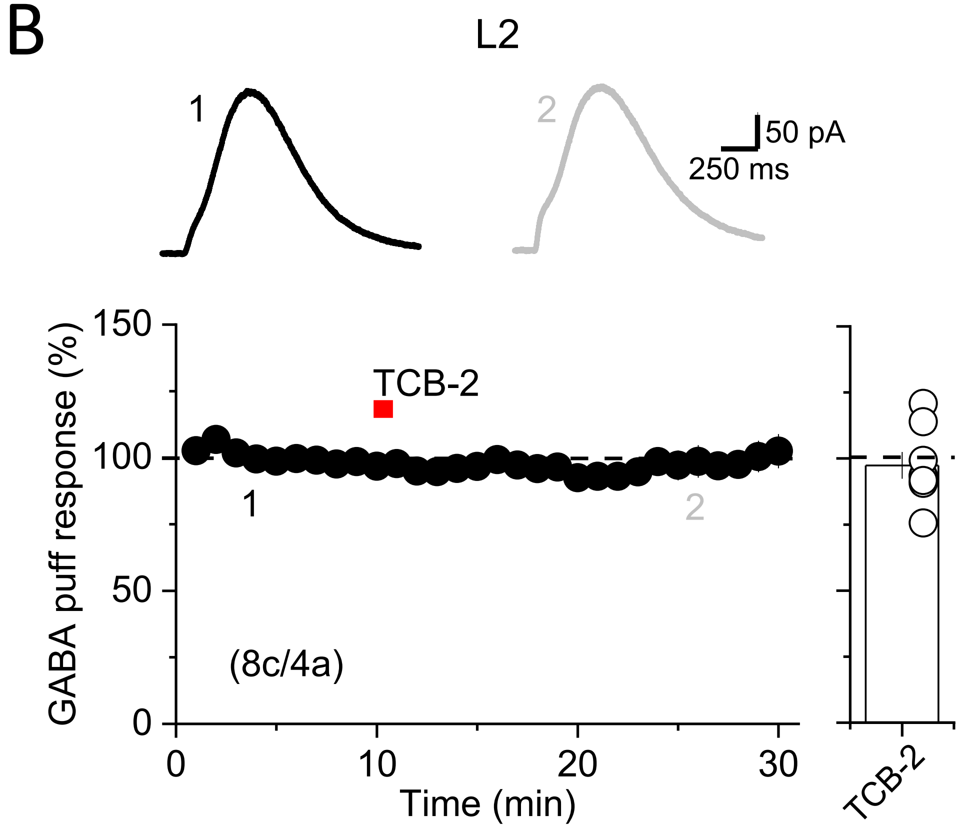

Supplementary Figure 5

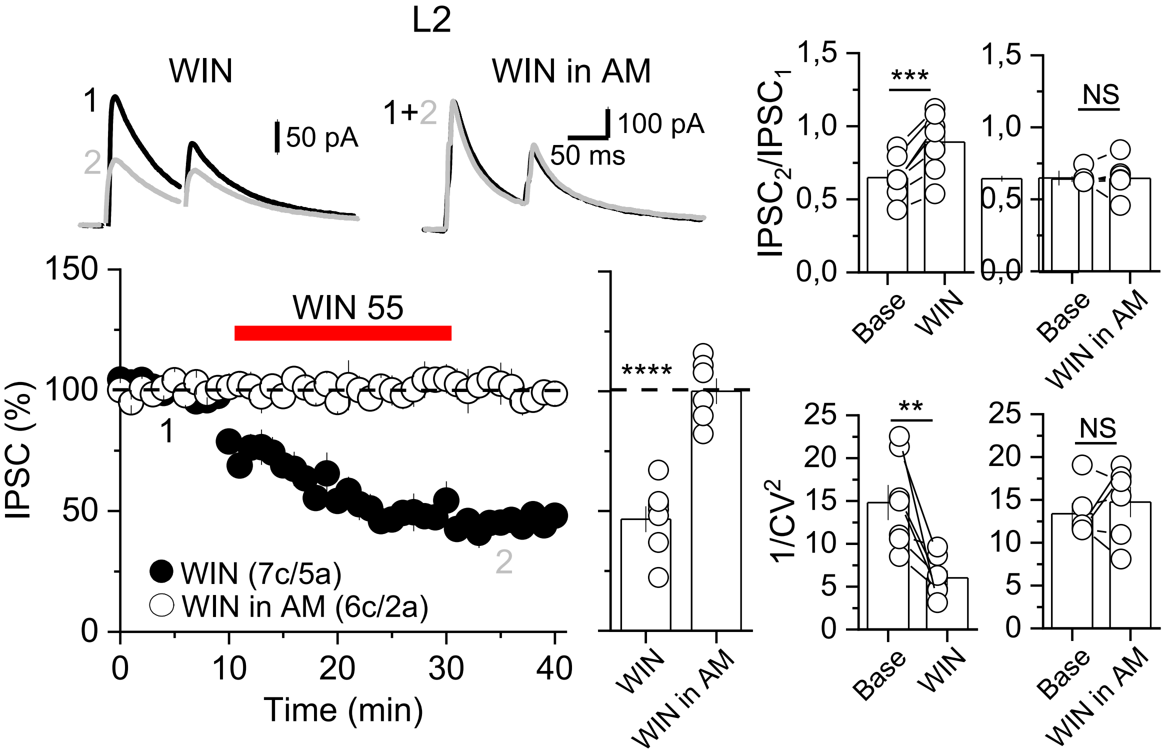

Supplementary Figure 6

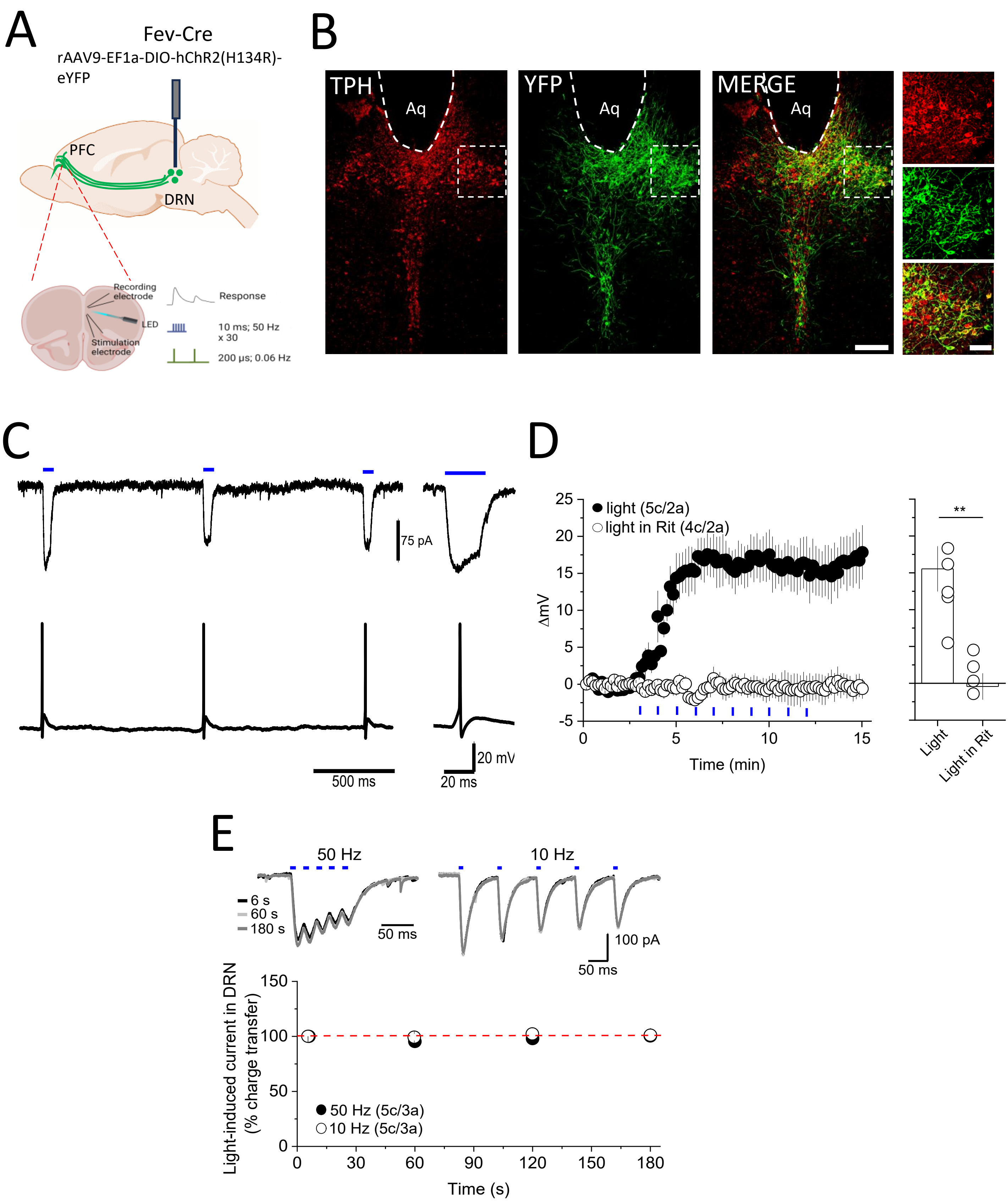

Supplementary Figure Legend

Supplementary Figure 1. (A) Representative traces and summarizes plots showing that bath application of 5-HT (50 μM) reduces the frequency but not the amplitude of spontaneous GABAergic IPSCs. **(B)** GABA-induced current evoked by puffing GABA (1 mM) directly on PNs in layer 2/3 was unaffected by adding 5-HT (50 μM). The number of cells (c) and animals (a) are indicated in parenthesis. ***P<0.001. NS, not significant.

Supplementary Figure 2. (A) Representative traces and summarizes plots showing that 5-HT-mediated depression of eIPSCs in layer 5 of the mPFC was barely reduced by either 5-HT1R antagonist cyanopindolol (Cya, 5 μM; *black dots*) or the 5-HT2R antagonist ritanserin (Rit, 4 μM; *open dots*) but was eliminated when both antagonists were applied together (*grey dots*). 5-HT-induced depression of eIPSCs in Cya or Rit was accompanied by changes in paired-pulse ratio (PPR; *top panel*) and the coefficient of variation (1/CV2; *bottom panel*). **(B)** Similar results were obtained in layer 2/3 using the 5-HT1AR blocker WAY100635 (1 μM; *black dots*) or the 5-HT2AR blocker MDL100907 (0.2 μM; *open dots*). **(C)** Comparative analysis showing the effect of Cya and Rit on 5-HT-mediated depression of eIPSCs in layer 2/3 (*data obtained from Figure 1C*) and layer 5 of the mPFC. Data are presented as mean ± SEM, and averaged sample traces taken at times indicated by numbers are shown next to each summary plot. The number of cells (c) and animals (a) are indicated in parenthesis. **P* <0.05, ***P* <0.01, ****P* <0.001.

Supplementary Figure 3. (A) Representative traces and summarizes plots showing that CP-93129 (3 μM), a specific 5-HT1BR agonist, had no effect on eIPSC in layer 2/3 and layer 5 of the mPFC. **(B)** CP-93129 (3 μM), however, significantly depresses perforant path to CA1 synapses in the hippocampus, an effect that was accompanied by changes in PPR and $1/CV^2$ and was eliminated in the continuous presence of the 5-HT1BR antagonist NAS-181 (6 μM). **(C)** Bath application of 5-HT (50 μM) depresses eIPSCs even in the presence of 5-H1BR antagonist NAS-181 (6 μM). **(D)**, Bath application of the selective 5-HT2CR agonist WAY161503 (3 μM) had no effect on eIPSC in layer 2/3 of the mPFC. Data are presented as mean ± SEM, and the number of cells (c) or slices (s) and animals (a) are indicated in parenthesis. **P* <0.05, ***P* <0.01. NS, not significant.

Supplementary Figure 4. (A) Representative traces and summarizes plots showing that TCB-2, but not 8-OH DPAT-induced depression of eIPSCs was eliminated when layer 2/3 PNs were loaded with the G protein blocker, GDPβS (2 mM). **(B)** Representative traces and time course showing that GABA-induced current, by puffing GABA (1 mM) directly on PNs, was unaffected by adding the 5-HT2AR agonist TCB-2 (3 μM). Data are presented as mean ± SEM, and the number of cells (c) and animals (a) are indicated in parenthesis. **P* <0.05, ***P* <0.01, ****P* <0.001, *****P* <0.0001. NS, not significant.

Supplementary Figure 5. Representative traces and summarizes plots showing that bath application of the CB1R agonist WIN 55,212 (WIN, 5 μM) significantly reduced eIPSC in layer 2/3 of the mPFC, an effect that was accompanied by changes in PPR and $1/CV^2$ and was eliminated in the continuous presence of the CB1R inverse agonist AM251 (AM, 4

μM). Data are presented as mean ± SEM, and the number of cells (c) and animals (a) are indicated in parenthesis. **P <0.01, ***P <0.001, ****P <0.0001. NS, not significant.

Supplementary Figure 6. (A), Schematic of virus injection in the dorsal raphe nucleus (DRN) of Fev-Cre mice and the protocol used to induce 5-HT-mediated depression of GABAergic synaptic response and LTD in the mPFC. **(B),** Confocal images of DRN neurons expressing ChR2-YFP (*green label*) and its colocalization with the enzyme tryptophan hydroxylase (TPH; *red label*). Scale bar: 200 μm and 100 μm for high and low magnification images, respectively. **(C)** DRN neurons expressing ChR2 depolarized by a light stimulation pulse (50 ms) when voltage clamped at -60 mV (*top*) and generated an action potential (*bottom*) in current clamp mode using K^+-methasulfonate-based internal solution (*see methods*). **(D),** Light-induced 5-HT release in the mPFC depolarized layer 2/3 PNs, an effect that was eliminated in naïve slices pre-incubated in the 5-HT2R antagonist ritanserin (Rit, 4 μM). **(E),** Representative traces and summarizes plot showing that light-induced current in ChR2-expressing DRNs during 50 Hz or 10 Hz stimulation protocol does not change the charge transfer over the time used to induced LTD. Data are presented as mean ± SEM, and the number of cells (c) and animals (a) are indicated in parenthesis. **P <0.01
